# Physicochemical characteristics and immunoregulatory activities of polysaccharides from five cultivars of Chrysanthemi Flos

**DOI:** 10.1002/fsn3.2720

**Published:** 2022-04-24

**Authors:** Ying Wang, Xuetao Chen, Ping Zhao, Lu Ren, Xia Li, Wenyuan Gao

**Affiliations:** ^1^ 117763 Taiyuan University of Science and Technology Taiyuan China; ^2^ 12605 Tianjin Key Laboratory for Modern Drug Delivery and High‐Efficiency Tianjin University Tianjin China; ^3^ 66353 Department of Chemistry Xinzhou Teachers University Xinzhou China

**Keywords:** chrysanthemums, immunomodulatory activities, physicochemical characteristics, polysaccharides

## Abstract

This study compared the physicochemical characteristics and immunomodulatory activities of chrysanthemums’ polysaccharides (JPs) from five cultivars. Significant differences were found in the molecular weights, the ratios of monosaccharide compositions, and morphological properties. Polysaccharides of Gongju (GJP) had the lowest molecular weight populations and polysaccharides of Boju (BJP) had the highest. *SEM* showed that GJP and polysaccharides of Qiju had looser and uniform surface structures, which are beneficial for being developed into instant products. Immunoregulatory assay revealed that JPs enhanced the phagocytosis and proliferation of RAW264.7 cells without obvious cytotoxicity, and upregulated the release level of TNF‐*α*, IFN‐*γ*, and NO. Immune‐enhancing activity correlated with their molecular weights, the contents of glucuronic acid and arabinose, and microstructure, which performed differently according to different cultivars. The results suggested that BJP and polysaccharides of Hangbaiju are more suitable to be developed as new functional foods for enhancing immunity, and provided a reference for selection based on the immunization requirements.

## Graphical abstract

Properties of five chrysanthemums’ polysaccharides were compared. Differences focused on the Mw, monosaccharide compositions, types, and morphology. HBJP and BJP have more potency be developed as immune pharmaceuticals.

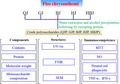

## 1 INTRODUCTION

Tea is the second largest consumed drink after water worldwide. The flower head of the *Chrysanthemum morifolium* Ramat. is widely consumed as tea owing to the wonderful flavor, color, and acclaimed health benefits (Wang et al., 2019). Chrysanthemum is a functional food and herbal medicine to cure fever, sore throats, and headache (Liang et al., 2014; Lin et al., 2015). The small molecules, such as phenols, flavonoids, alkanes, terpenoids, and unsaturated fatty acids, are often in charge of its bioactivities, and the compositions are varied obviously from different cultivars, geographic variations, and harvest time of chrysanthemum (Wang et al., 2015; Yuan et al., 2015). So far, present researches on chrysanthemum mainly focused on these small‐molecule compounds and their bioactivities as well as extraction technology (Yuan et al., 2015). Five major cultivars in market circulation, including Hangbaiju (Zhejiang province), Boju and Gongju (Anhui province), Huaiju (Henan province), and Qiju (Hebei province), classified by their origins, are well known in Asia and Europe for more than two thousand years (Yuan et al., 2015). Meanwhile, chrysanthemum is one of the most important economic crops in China which is currently the world's largest producer and exporter of chrysanthemum. It is widely consumed by producing tea, wine, cakes, porridge, condiment, and is used as a dietary supplement.

Polysaccharides are one of the dominant substances in hot water extracts, and the water‐soluble polysaccharides are a group of active components promising immunocompetence with little toxicity (Yang et al., 2017). Natural carbohydrates are abundant in chrysanthemum, but unlike the sufficient studies about low‐molecule active substances, less reports focused on polysaccharides from chrysanthemum. Yuan et al. (2019) compared the monosaccharide composition and molecular weight of polysaccharides extracted from *Coreopsis tinctoria* (snow chrysanthemum), *Chrysanthemum indicum*, “Huangju,” “Gongju,” and “Hangbaiju.” The polysaccharides exhibited remarkable antioxidant and antiglycation activities. Zhang et al. (2019) extracted a novel arabinogalactan from snow chrysanthemum, which possessed a 1,6‐linked β‐D‐Galp and 1,5‐linked α‐L‐Araf backbone. The inhibitory effects on α‐amylase and α‐glucosidase were 2.7 and 17.9 times that of acarbose, respectively. The study of digestion in vitro and fecal fermentation suggested that snow chrysanthemum polysaccharides (JHP) could be partially degraded under the gastrointestinal digestion and further consumed by gut microbiota. JHP could increase beneficial bacteria significantly (Wu et al., 2021). The primary therapeutic effect of chrysanthemum used in herbal medicine is heat‐clearing and detoxifying, specifically anti‐influenza virus, involving macrophage immunodeficiency. Previous reports showed that polysaccharides from Huaiju and Hangbaiju possess antitumor effect (Zheng et al., 2015), as well as polysaccharides from Boju can stimulate the proliferation of T and B lymphocyte via inducing the production of mitogen (Zheng et al., 2006). The bioactivities of polysaccharides can be significantly affected by their physicochemical properties, including molecular size, ratios of constituent monosaccharides, glycosidic linkages, and the chain conformations (Gong et al., 2015; Su et al., 2016). The physicochemical characteristics and the immune bioactivity of chrysanthemum's polysaccharides (JPs) may be influenced by cultivars and geographical environment, which are worthy of systematic comparative research. Meanwhile, selecting chrysanthemum plantations for better application in food and drug industry from the perspective of immunization requirements is very useful.

In this study, physicochemical properties and immunostimulatory activities of polysaccharides from five cultivars of chrysanthemum were compared. The effects of the five polysaccharides’ water solution on RAW264.7 cells’ reproductive capacity, phagocytosis, and release levels of inflammatory cytokines were tested. These results provide detailed information for polysaccharides in chrysanthemums from different cultivars, including the variation in polysaccharides’ physicochemical properties and immunostimulatory activities. This study would supply chrysanthemum's polysaccharides with high immunocompetence that can be potentially used in the manufacture of a new functional food and pharmaceutical.

## 2 MATERIALS AND METHODS

### 2.1 Plants and reagents

The fresh flower of Qiju was gathered from Anguo, Hebei province; Hangbaiju was gathered from Jiaxing, Zhejiang province; Boju was collected from Bozhou and Gongju was gathered from Huangshan, Anhui province; and Huaiju was collected from Jiaozuo, Henan province. The specimens had been identified by Professor Wenyuan Gao and stored in the School of Pharmaceutical Science and Technology, Tianjin University, China. The detailed information of their growing environment is given in Table S1. The flower head of chrysanthemum (each about 1000 g) was collected in November of 2017. Freshly collected material was brought back to the laboratory in one day and stored at 4°C, and dried on the second day. Samples collected from each natural population were oven dried at 75°C to constant weight, and stored at room temperature for the subsequent analysis.

Bovine serum albumin (BSA), Coomassie brilliant blue G250, dextrans, mannose (Man), rhamnose (Rha), galacturonic acid (GalA), glucuronic acid (GlcA), glucose (Glc), xylose (Xyl), arabinose (Ara), galactose (Gal), 1‐phenyl‐3‐methyl‐5‐pyrazolone (PMP), 3‐(4,5‐dimethylthiazol‐2‐y1)‐2,5‐ diphenyltetrazolium bromide (MTT), and lipopolysaccharide (LPS) were provided by Sigma‐Aldrich Chemical Co. The RAW264.7 macrophage cell lines, ELISA test kits for tumor necrosis factor *α* (TNF‐*α*) and interferon *γ* (IFN‐*γ*), and Griess reagent kits were acquired from the MLbio Biotechnology Co. Ltd. Dulbecco's Modified Eagle's Medium (DMEM) and fetal bovine serum (FBS) were supplied by Gibco Invitrogen Co. HPLC‐grade acetonitrile was supplied by Concord Technology Co. Ltd., and the rest of reagents were analytically pure and purchased from Tianjin Jiangtian Chemical Technology Co., Ltd.

### 2.2 Extraction of polysaccharides from chrysanthemum

The polysaccharides were acquired on the basis of the previous method (Wang et al., 2019) with minor modification. The dried samples (50 g) were cut into pieces and refluxed with ethanol (500 ml, 75°C) twice for 4 h to remove liposoluble constituents and pigments, and then filtered. Each residue was refluxed for 4 h with ten times of deionized water at 75°C for twice. The filter liquor was obtained by suction filtration through a filter paper on a Buchner funnel and concentrated with a vacuum rotary evaporator at 60°C to 200 ml, followed by removing of the free proteins from the extracts using the Sevage reagent (chloroform:n‐butanol = 4:1, v/v). After the organic solvent was removed, polysaccharides were collected by precipitating with 800 ml anhydrous ethanol and maintained overnight at 4°C. The precipitate was gathered by centrifugation (3,000 g, 10 min) and was redissolved and dialyzed against distilled water for 48 h with water change every 4 h (molecular weight cut‐off 8–14 kDa). The solution was lyophilized to acquire samples of polysaccharides. The polysaccharides obtained from “Boju,” “Gongju,” “Huaiju,” “Qiju,” and “Hangbaiju” were named “BJP,” “GJP,” “HJP,” “QJP,” and “HBJP,” respectively.

### 2.3 Preliminary characterizations of polysaccharides

#### 2.3.1 Determination of polysaccharides and proteins

The total polysaccharides (TP) were measured using phenol–sulfuric acid method and glucose acted as standard substance to establish the standard curve. The protein content in the polysaccharides was measured using Coomassie brilliant blue method and BSA was used as standard (Zhao et al., 2017).

#### 2.3.2 UV–vis spectroscopy

UV–vis spectra of polysaccharides water solution were obtained via a Cary 60 UV–visible spectrophotometer (Agilent, USA) within 190–400 nm.

#### 2.3.3 Molecular weight evaluation

Molecular weights (Mw) were measured on the basis of the previous method with minor modification (Wang et al., 2019). An HPLC (Shimadzu, Kyoto, Japan) system with a gel‐filtration chromatography column of TSK gel GMPW_XL_ column (7.8 mm × 300 mm, Tosoh Corp., Japan) and an evaporative light scattering detector (ELSD) was used. The flow rate of carrier gas was controlled at 2.2 L/min and the temperature of drift tube was 70°C. The polysaccharides’ water solution (5 mg/ml) were filtered through a 0.22‐μm filter film and 30 μl of samples was injected. The column temperature was controlled at 30°C, and each sample was eluted with ultrapure water (0.5 ml/min). The specification curve was constructed with sequentially increased Mw of dextrans (5, 12, 50, 150, 210, and 410 kDa).

#### 2.3.4 Monosaccharide constitution

Each sample (4 mg) was first dissolved in trifluoroacetic acid (2 ml, 2 M) and then hydrolyzation was carried out at 120°C for 6 h in a sealed ampoule, and dried by a vacuum rotary evaporator at 60°C. Methanol (2 ml) was added and codistillated to eliminate the unreacted trifluoroacetic acid, and this treatment was repeated three times. Subsequently, the hydrolysates (200 μl) and monosaccharide standards (200 μl, 2 mg/ml) were performed with derivatization procedure. NaOH (50 μl, 0.5 M) and methanolic solutions of PMP (50 μl, 0.5 M) were added, and maintained for 30 min at 70°C reducing sugar to sugar alcohol. After cooling, 50 μl of HCl (0.3 M) was added to neutralize the residual NaOH and ultrapure water of 1 ml was added. Chloroform (1 ml) was added to extract and abandon the excess PMP. Multiplicating this process, the water solution was passed through a 0.22‐μm syringe filter to analyze monosaccharide constitution, and a Shimadzu HPLC equipped with a photodiode array detector (PAD) was used. PMP‐derived samples (10 μl) were applied to a kromasil 100–5 C18 column (4.6 mm × 250 mm, 5 μm) at 30°C and the UV monitor was set at 250 nm. Acetonitrile and ammonium acetate buffer (20 mM, pH 5.0) with a volume ratio of 22:78 were used as mobile phase and the flow rate was controlled at 1.0 ml/min. The monosaccharides were confirmed by the standards (Figure S1) and the ratio was calculated by their peak areas.

#### 2.3.5 FTIR spectral analysis

The dry powder samples (2 mg) were ground and tablet with KBr (200 mg) was prepared. The FTIR spectrum of each sample was detected on a Tensor 27 spectrometer (Bruker Optics Inc.) and recorded from 4000 to 400 cm^−1^.

#### 2.3.6 Morphological properties

The shape, size, and surface properties of JPs were recorded using a field emission *SEM* (NOVA Nanosem 430, FEI Company). The JPs were adhered to a specimen holder and sputtered with gold for 70 s at 20 mA. Each sample was scanned with an accelerating voltage at 20 kV.

### 2.4 Immune‐enhancing activity analysis

#### 2.4.1 RAW264.7 cell viability assay

The MTT assay was performed to evaluate the RAW264.7 cell proliferation when treated with three concentrations of JPs water solution (12.5, 50, and 200 µg/ml) (Bi et al., 2018). RAW264.7 cells were preincubated in a 96‐well microplate with 1 × 10^5^ cells per well (100 µl) for 24 h and JPs water solution (100 µl) with three concentrations were used to treat. The positive control was treated with lipopolysaccharide (LPS, 50 µg/ml). After continuous culturing for 48 h, 50 µl of 2 mg/ml MTT was appended followed by another cultivation for 4 h. Dimethyl sulfoxide (100 µl) was appended to dissolve the precipitate after removing the medium. Cell viability was evaluated by determining the absorption at 570 nm using an Infinite M200 PRO spectrophotometer (Tecan Co.).

#### 2.4.2 Phagocytosis assay

The determination of cells’ phagocytic ability was performed following the method of Neutral red uptake reported by Bi et al. (2018). RAW264.7 cells (100 µl, 1 × 10^6^ cells/ml) were cocultured with 100 µl of different concentrations of JPs (12.5, 50, and 200 µg/ml) in 96‐well plates for 48 h. The positive control was treated with LPS (50 µg/ml) and the blank control was treated with medium. The unattached cells were rinsed by PBS twice followed by adding neutral red solution (100 µl). The supernatant was abandoned after further incubating for 1 h. PBS solution (200 µl) was used to wash the neutral red solution that was not engulfed twice, and then cell lysate (100 µl, 1 M acetic acid and ethanol in a 1:1 volume ratio) was added. After incubation overnight at 37°C, the value of OD570 nm was detected.

#### 2.4.3 Determination of the release of TNF‐α and IFN‐γ

RAW264.7 cells were cultured in a 96‐well plate for 24 h (100 µl, 1 × 10^6^ cells/ml), and incubated with 100 µl of three concentrations of JPs (12.5, 50, and 200 µg/ml) and LPS (50 µg/ml) for 48 h. The concentrations of TNF‐*α* and IFN‐*γ* were measured using ELISA kits in accordance with the manufacturer's instructions.

#### 2.4.4 Measurement of the production of NO

Cells were planted onto a 96‐well plate for 24 h (100 µl, 1 × 10^6^ cells/ml) and 100 µl of different concentrations of polysaccharides (12.5, 50, and 200 µg/ml) was added, followed by further 48‐h culturing. The positive control was treated with LPS (50 µg/ml) and the solvent control was treated with double‐distilled water. The NO content in the supernatants was detected by the Griess reagent.

### 2.5 Statistics

All experiments were carried out for three times. One‐way analysis of variance (ANOVA) was carried out to test significant differences (*p* < .05) with S‐N‐K and LSD *post hoc* test was carried out to analyze univariate comparisons by IBM SPSS 20.0.

## 3 RESULTS AND DISCUSSION

### 3.1 The yield of the crude polysaccharides content, polysaccharides, and protein content

**FIGURE 1:**
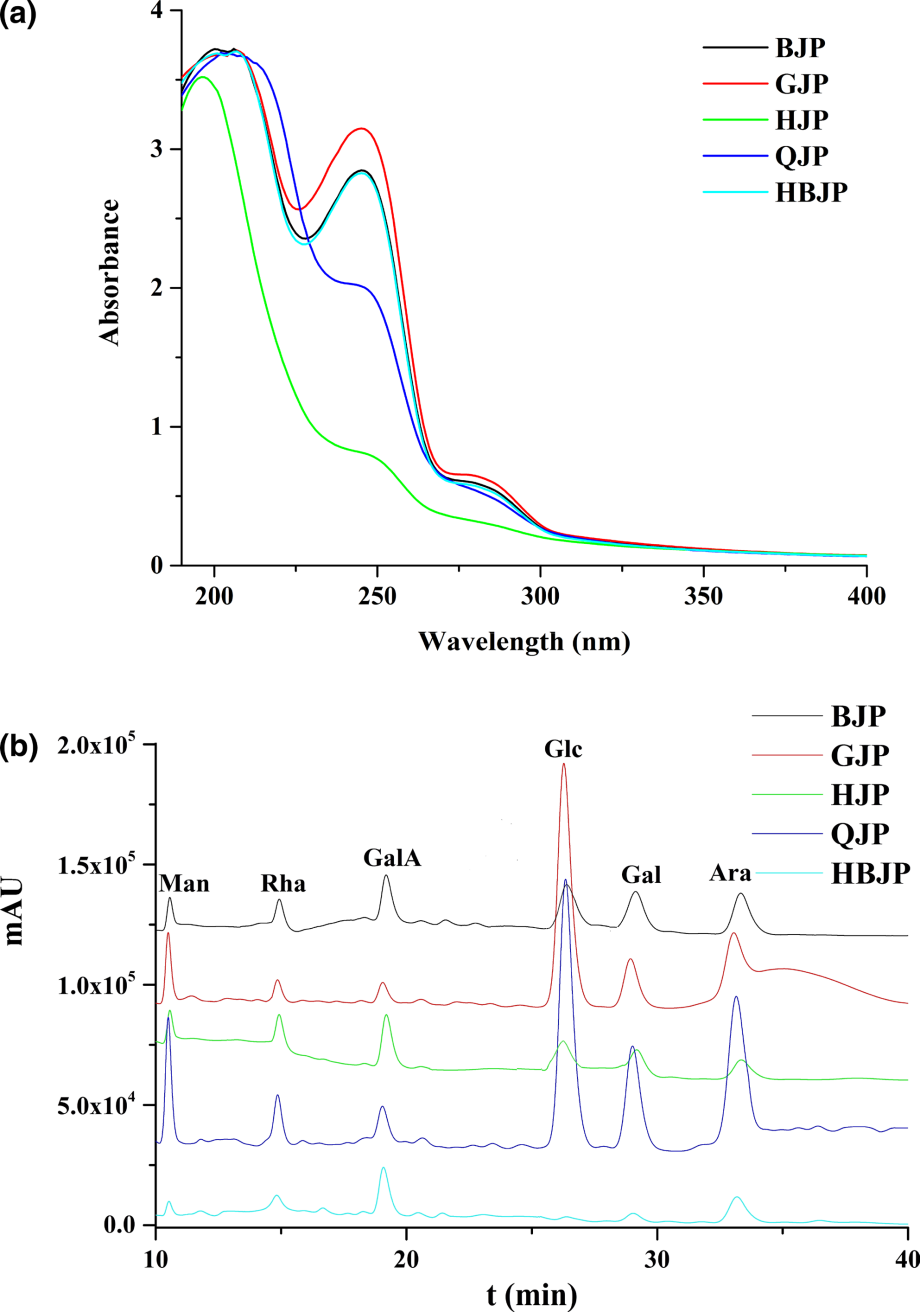
(a) UV spectra, (b) Monosaccharide composition analysis of BJP, GJP, HJP, QJP, and HBJP

It turned out that the yield of crude polysaccharides ranged from 6.43% (QJP) to 11.05% (HBJP), as shown in Table 1, which was higher than that of Yuan et al.’s (2019) study (3.2%–7.3%). The content of polysaccharides from JPs ranged from 51.45% to 60.47%, which was lower than 78.8%–89.2% (Yuan et al., 2019). BJP contained markedly higher level than others (*p* < .05). The existence of protein was confirmed by UV absorption peaks at 280 nm (Figure 1a) and the content was determined by Coomassie brilliant blue, as well as the peak value was detected in GJP (7.48%). The absorption at 260 nm is characteristic for nucleic acid, implying that the JPs containing nucleic acid and the peak value were detected in GJP. Based on the yield of the crude polysaccharides content, total polysaccharides, and protein contents, HBJP and BJP can be recommended for chrysanthemum polysaccharide resource in the industrial manufacture.

**TABLE 1:** The yield, total polysaccharides, protein, molecular weight, and monosaccharide composition of BJP, GJP, HJP, QJP, and HBJP

Samples	HJP	QJP	HBJP	BJP	GJP
Yield (%)	8.11	6.43	11.05	9.53	7.51
TP (%)	51.45 ± 2.40^b^	53.42 ± 1.85^c^	54.69 ± 1.74^c^	60.47 ± 2.01^d^	49.48 ± 1.81^a^
Protein (%)	6.02 ± 0.59^a^	6.30 ± 0.34^a^	7.16 ± 0.24^b^	7.14 ± 0.42^b^	7.48 ± 0.45^b^
Mw (kDa)	2106.09	1179.38	1000.70	2391.36	60.08
	412.82	204.50	210.46	40.33	8.52
	59.78	73.13	91.51		2.29
Sugar composition					
Man (%)	11.82 ± 0.40^c^	7.43 ± 0.83^b^	3.42 ± 0.11^a^	3.82 ± 0.30^a^	6.86 ± 0.36^b^
Rha (%)	4.36 ± 0.23^b^	4.56 ± 0.16^b^	23.30 ± 1.02^c^	2.99 ± 0.15^a^	4.22 ± 0.21^b^
GalA (%)	9.87 ± 0.15^c^	4.79 ± 0.32^a^	34.14 ± 1.24^d^	6.79 ± 0.21^b^	4.61 ± 0.13a^b^
Glc (%)	60.08 ± 2.49^d^	36.09 ± 0.25^b^	7.53 ± 0.46^a^	66.63 ± 2.93^e^	47.81 ± 2.17^c^
Gal (%)	7.53 ± 0.22^a^	14.89 ± 0.19^d^	8.87 ± 0.41^b^	9.73 ± 0.51^c^	10.37 ± 0.22^c^
Ara (%)	6.34 ± 0.45^a^	32.25 ± 2.13^e^	22.74 ± 2.06^c^	10.04 ± 0.95^b^	26.14 ± 1.96^d^

Note

Different letters within the same substance show significant difference at *p* < .05. The results are expressed as means ± *SD* (*n* = 3).

### 3.2 Molecular weights

The standard curve of Mw is shown in Figure S2. The polysaccharides extracted from chrysanthemums presented wide and dispersive molecular size distribution, as shown in Table 1 and Figure S3. There were three distinct macromolecular populations with the peak Mw of 2106.09, 412.82, and 59.78 kDa for HJP. QJP (1179.38, 204.50, and 73.13 kDa), GJP (60.08, 8.52, and 2.29 kDa), and HBJP (1000.70, 210.46, and 91.51 kDa) showed three groups. Two major groups were determined in BJP (2391.36 and 40.33 kDa). In the previous study, two macromolecular populations were found in GJP (530 and 41.1 kDa) and HBJP (588 and 45.5 kDa) (Yuan et al., 2019). The values of Mw could be affected by the samples and experimental conditions in different papers. The results demonstrated that the polysaccharides of chrysanthemums from different cultivars are all diverse Mw populations and heterogeneous.

### 3.3 Monosaccharide analysis

The monosaccharide components in polysaccharides from diffident chrysanthemums were similar, which comprised Rha, Ara, GalA, Man, Glc, and Gal with various ratios (Figure 1b). The results showed that HJP and BJP were abundant in Glc (60.08% and 66.63%) (Table 1), while Glc and Ara were the predominant monosaccharide in GJP (47.81% and 26.14%) and QJP (36.09% and 32.25%). HBJP was rich in Rha, GalA, and Ara (23.30%, 34.14%, and 22.74%) and a higher content of uronic acid might possess a higher bioactivities according to previous reports (Ma et al., 2013; Wang et al., 2019; Zhao et al., 2017). In addition to the gene, precipitation, light radiation, and temperature of the planting areas were different according to the geographic variations, which would influence the content of secondary metabolites and the polysaccharide accumulation (Liu et al., 2018). In this study, it was speculated that the monosaccharide compositions were influenced by environmental characteristics.

### 3.4 FTIR

**FIGURE 2:**
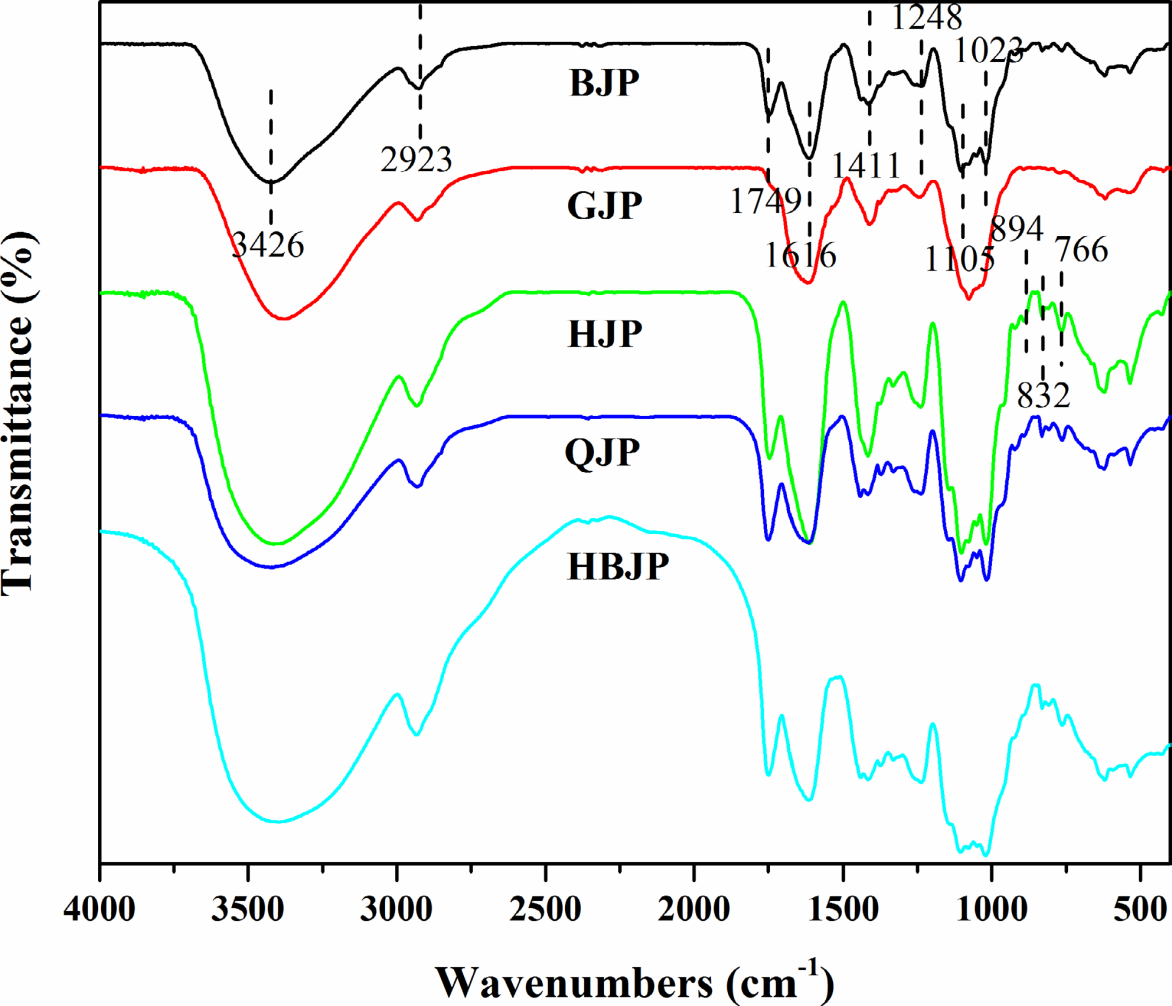
FT‐IR spectra of BJP, GJP, HJP, QJP, and HBJP

The FTIR spectra of five polysaccharides are shown in Figure 2, and they seem similar. The absorption peak centered at 3426 cm^−1^ corresponded to hydroxyl groups. The band centered at 2923 cm^−1^ corresponded to the C‐H asymmetric extensional vibration of the CH_2_ or CH_3_. The peak at 1749 cm^−1^ was assigned to the absorption of C = O, which indicated the presence of uronic acids (Cao et al., 2018). The peak at 1616 cm^−1^ was characteristic for the protein secondary structural components, meaning that conjugated proteins existed, and the peak at 1411 cm^−1^ was characteristic for the C‐H variable angle vibration. A series of bands at 1000–1200 cm^−1^ corresponded to the vibrations of C‐O, C‐C, and C‐OH. The peak centered at 894 cm^−1^ was characteristic of *β*‐glycosidic linkages and 832 cm^−1^ showed the presence of *α*‐glycosidic linkages (Zhao et al., 2017).

### 3.5 SEM

**FIGURE 3:**
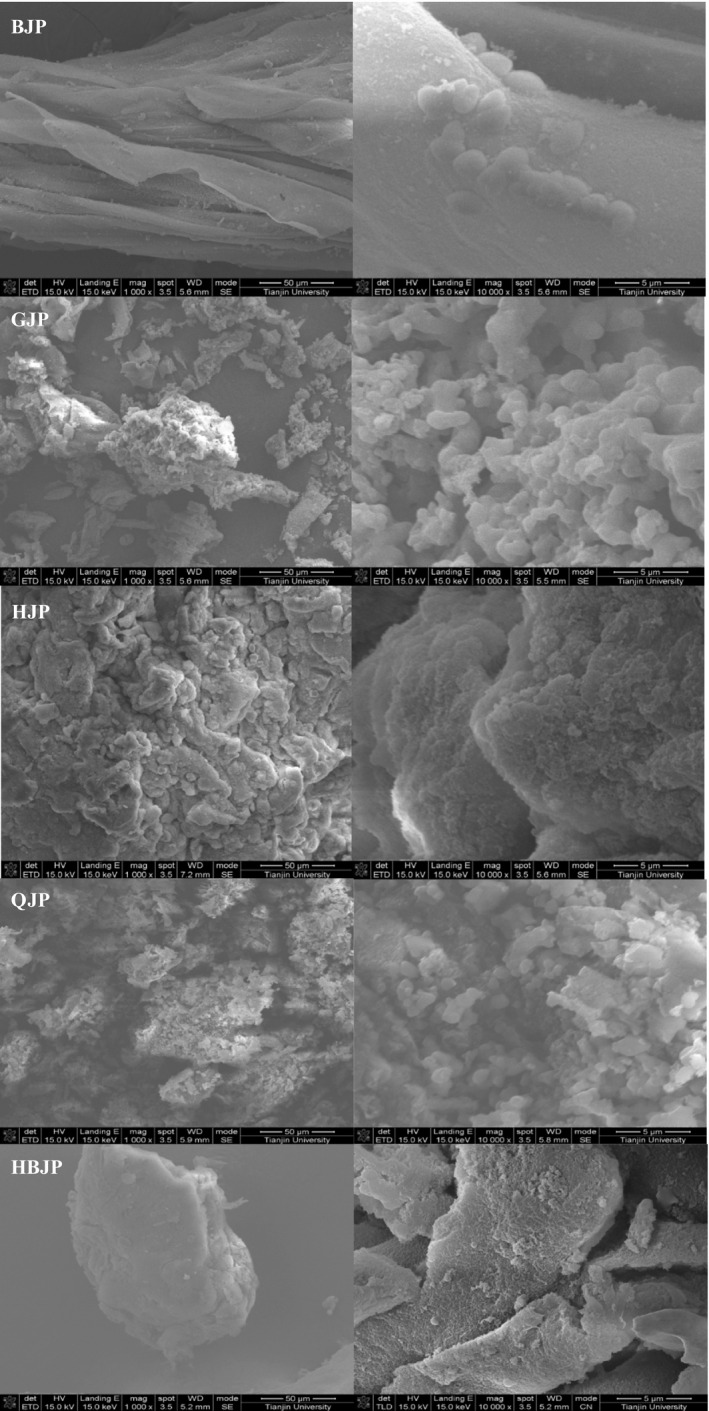
SEM of BJP, GJP, HJP, QJP, and HBJP (left: ×1000; right: ×10,000)

The morphological properties of JPs exhibited differences in shape, size (1000×), and surface features (10,000×), and the size of the particle was positive with Mw roughly (Figure 3). In general, HJP, GJP, HBJP, and QJP presented particles with the uneven size of block structure. HJP was aggregation by more loose structure with some porosity on surface, while HBJP has a compact structure with large cracks. GJP owns the smallest particle size (1000×) and is made up of a uniform collection of particles, which was similar to the apparent structure of QJP. BJP possessed accumulated thin layer structure and small globular particles attach. The morphological properties of polysaccharides are important for the reconstitution of the powder products, which affect the solubility of the final products (Wang et al., 2019). Therefore, a smaller particle size, more uniform, and loose structure of GJP and QJP were beneficial when used in food and medicine industry than the compact configuration of HJP and HBJP.

### 3.6 Immune‐enhancing activity of JPs

Previous studies observed that polysaccharides administered orally can activate the immune system in the small intestine (Bi et al., 2018). The immunomodulatory effect of polysaccharides is mainly through the stimulation of multi‐effector cells (Tang et al., 2018). Macrophages participate in nonspecific and specific immunity in vivo, playing a vital role via increasing phagocytic activity, activation of lymphocyte, and promoting the secretion of proinflammatory factors, for instance, TNF‐*α*, IFN‐*γ*, as well as secretion nitric oxide (NO) by inhibiting the activation of transcription factors (NF‐κB) (Bi et al., 2018; Cao et al., 2018). For polysaccharides, various receptors were specifically identified and bonded by different monosaccharides, which activate the cell signaling cascades. Activated receptors further improve adaptive immune responses through releasing of cytokines (Tang et al., 2018). Polysaccharides from various species exhibited obviously different characteristics in their biological activity. Molecular weight and monosaccharide composition are important physicochemical properties of polysaccharides, although not exclusive properties related to immune activity. The relationship between the immunomodulatory potency of the five chrysanthemums polysaccharides and their physicochemical differences can be inferred preliminarily according to references.

#### 3.6.1 RAW264.7 cell viability

**FIGURE 4:**
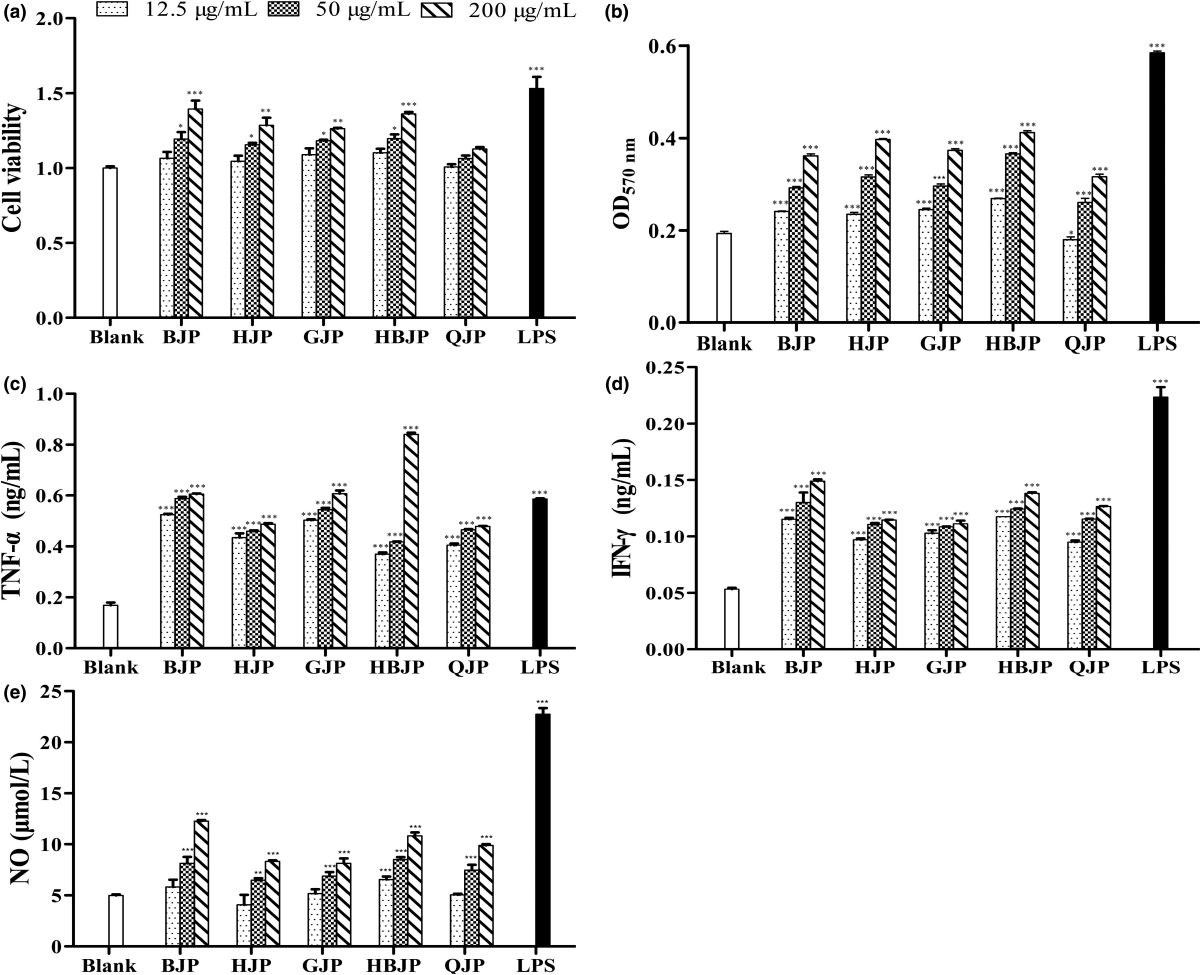
(a) Effect of JPs on vitality of RAW264.7 cells. (b) Effect of JPs on the phagocytic capacity. Effect of JPs on TNF‐α (c), IFN‐γ (d), and NO (e) release. *p < .05, **p < .01, ***p < .001 versus the blank control.

Macrophages are considered to be of vital importance to innate immune response and mediate host defense (Du et al., 2018). In order to estimate the impact of five sources of JPs on the viability of RAW264.7 macrophages, MTT assays were performed at three different concentrations (12.5, 50, and 200 µg/ml). JPs at low concentration did not affect the cell viability remarkably as shown in Figure 4a (*p* ≥ .05). BJP and HBJP at concentrations of 200 µg/ml increased cell viability significantly (*p* < .001). For QJP, no discernable difference was found among the three concentrations and the blank control (*p* ≥ .05). Unlike microorganisms polysaccharides and chemical drugs, most botanical polysaccharides do not exhibit obvious cytotoxicity and almost have no side effects (Li et al., 2018). Figure 4a shows that JPs (12.5–200 μg/ml) would not cause apoptosis of RAW264.7 cells, suggesting that they are effectively activating macrophages without toxic effects.

#### 3.6.2 Phagocytic capacity of RAW264.7 cell

Phagocytosis is one of the most basic defense systems in innate immunity, involved in interfering with the viral replication, distinguishing and phagocytosing foreign bodies, such as bacteria, viruses, damaged cells (Bi et al., 2018; Li et al., 2018). The results of neutral red uptake test indicated that JPs (12.5–200 μg/mL) observably stimulated the phagocytosis activity of RAW264.7 cells except the low concentration of QJP and the phagocytic capacity, depending on the concentration of samples (Figure 4b) (*p* < .001). Thus, a remarkable promotion of activated macrophages was observed after the treatment of JPs. It was reported that some botanical polysaccharides induced immunomodulatory responses mainly by specific recognizing and binding to various membrane receptors (Li et al., 2018). Ara, Gal, and Rha contained in polysaccharides from *Colocasia esculenta* can be recognized by toll‐like receptor‐2 (TLR‐2) and TLR‐4 in previous study (Li et al., 2018). Therefore, the high content of the sum of the three monosaccharides in HBJP made it own the best phagocytic capacity. Previous studies also reported that glucose contained in polysaccharides was recognized in scavenger receptor, glucocorticoid receptor, TLR‐2, and TLR‐4 (Chen et al., 2017). Therefore, a higher content of glucose in BJP and HJP was the dominant factor that led to a higher immune activity.

#### 3.6.3 Cytokine secretion of RAW264.7 cell

Cytokines, low molecular weight and soluble pleiotropic peptides or glycoproteins, can mediate the unleashing of an effective immune response, link innate and adaptive immunity, and influence the macrophage's microenvironment (Tang et al., 2018). TNF‐*α*, IFN‐*γ*, and NO are major proinflammatory cytokines that participate in inflammatory response and are in charge of antineoplastic, antiviral, and cytolytic functions (Bi et al., 2018). JPs can remarkably upregulate the secretion level of TNF‐*α* and IFN‐*γ* (*p* < .001), exhibiting an effective proinflammatory activity and the cytokine release level depending on the concentration of JPs (Figure 4c,d). The concentration of TNF‐*α* in RAW264.7 cells was determined as 0.40 ng/ml approximately at the low concentration of JPs and the highest reached 0.84 ng/ml at 200 µg/ml of HBJP. The peak value was higher than that of LPS. A higher released level of TNF‐*α* stimulated by HBJP was correlated to lower Mw, higher ratios of GalA and Ara (Cao et al., 2018; Fimbres et al., 2018). Compared with blank group, the secretion of IFN‐*γ* was also remarkably improved in RAW264.7 cells when treated with JPs (*p* < .001), and the peak value was obtained at 200 µg/ml of BJP. The others were in the following order roughly: HBJP > QJP > HJP > GJP.

NO is a signaling molecule related to macrophage cytolytic function, inflammatory response, and signal transduction regulation in the immune response (Yang et al., 2018; Zhang et al., 2012). The production level of NO in cells handled with JPs was also measured in this study. Results showed that treatment with a moderate and high dose of JPs could stimulate NO secretion significantly (*p* < .001) (Figure 4e). The effect of upregulation of NO in supernatants acts on killing microbes, parasites, and tumor cells (Bi et al., 2018). The significant improvement of NO secretion by JPs was mainly on account of the provocation of NO synthase expression and BJP owning the highest secretion level of NO.

Previous studies showed that plant polysaccharides containing uronic acids were valid in activating macrophages, and improving the release of immune‐related cytokines, as well as increasing the expression of immune‐related genes (Cao et al., 2018; Tang et al., 2018). According to the present results, the immune‐enhancing activity of BJP and HBJP is more intensified than others. The immune activity is closely related to polysaccharides’ physicochemical characteristics, for instance, Mw, water solubility, chemical compositions, glycosidic‐linkage, and degree of branching (Fimbres et al., 2018; Li et al., 2018; Wu et al., 2022). It was reported that arabinose contained in polysaccharides showed an immunomodulatory activity (Li et al., 2018). The disparate immunological activity between JPs mainly resulted from the variations in monosaccharide composition, according to the results of physicochemical properties and immunity test. The immunomodulatory potency of JPs variance depended on chrysanthemum's cultivar.

## 4 CONCLUSIONS

Physicochemical characteristics and immunostimulatory activities of the water‐soluble polysaccharides from five different cultivars of chrysanthemums varied significantly. The differences were mainly focused on Mw, monosaccharide composition ratios, and morphological properties. The results of immunostimulatory activities indicated that the polysaccharides of chrysanthemums possess immunomodulating activities, and could potentially be developed into immune foods and pharmaceuticals, especially HBJP and BJP. This study provided an option of consumption based on the need of immunization. GJP and QJP are good candidates for exploring as a functional food additive due to the loose and uniform structure. In short, the present study has supplied the essential information about the variances in physicochemical characteristics and immunization of polysaccharides from five edible and medicinal chrysanthemums.

## CONFLICT OF INTEREST

The authors confirm that they have no conflicts of interest with respect to the study described in this manuscript.

## Supporting information

Supinfo

## Data Availability

The data that support the findings of this study are available from the corresponding author upon reasonable request.
