# Supplementary material for: Physicochemical characteristics and immunoregulatory activities of polysaccharides from five cultivars of Chrysanthemi Flos
Source: Food Sci Nutr. 2022 Apr 24;10(5):1391–400. doi: 10.1002/fsn3.2720 (PMC9094477; doi:10.1002/fsn3.2720)
Supplement: Supplementary file 1 — Supinfo [file FSN3-10-1391-s001.pdf]

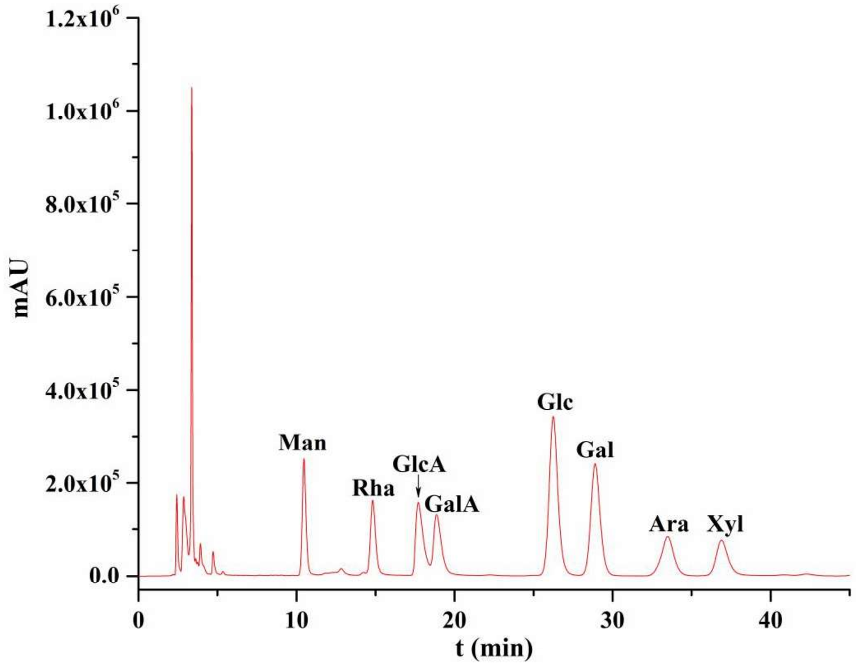

Fig. S1 HPLC for monosaccharide composition analysis (standard substances).

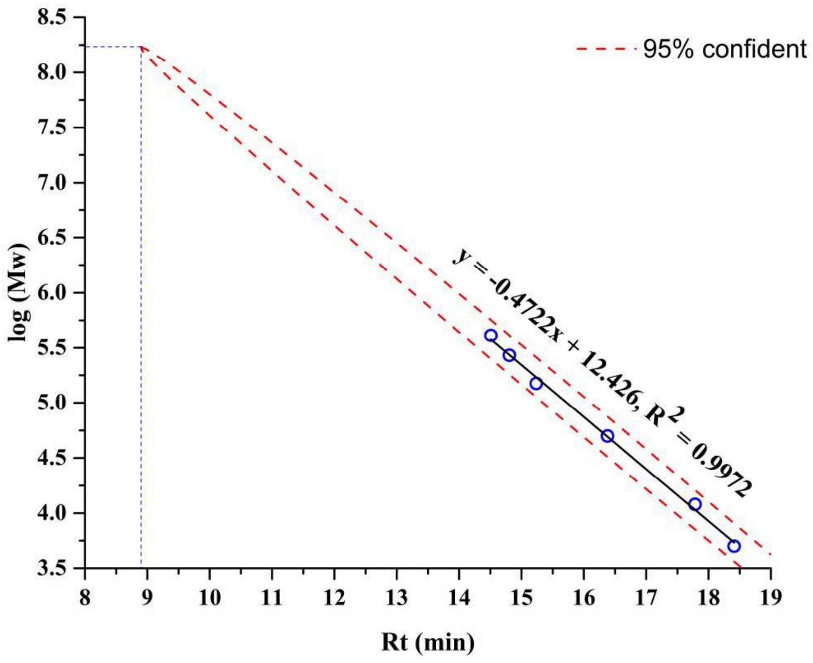

Fig. S2 A standard curve was constructed using dextrans with various molecular weight (5, 12, 50, 150, 210, and 410 kDa).

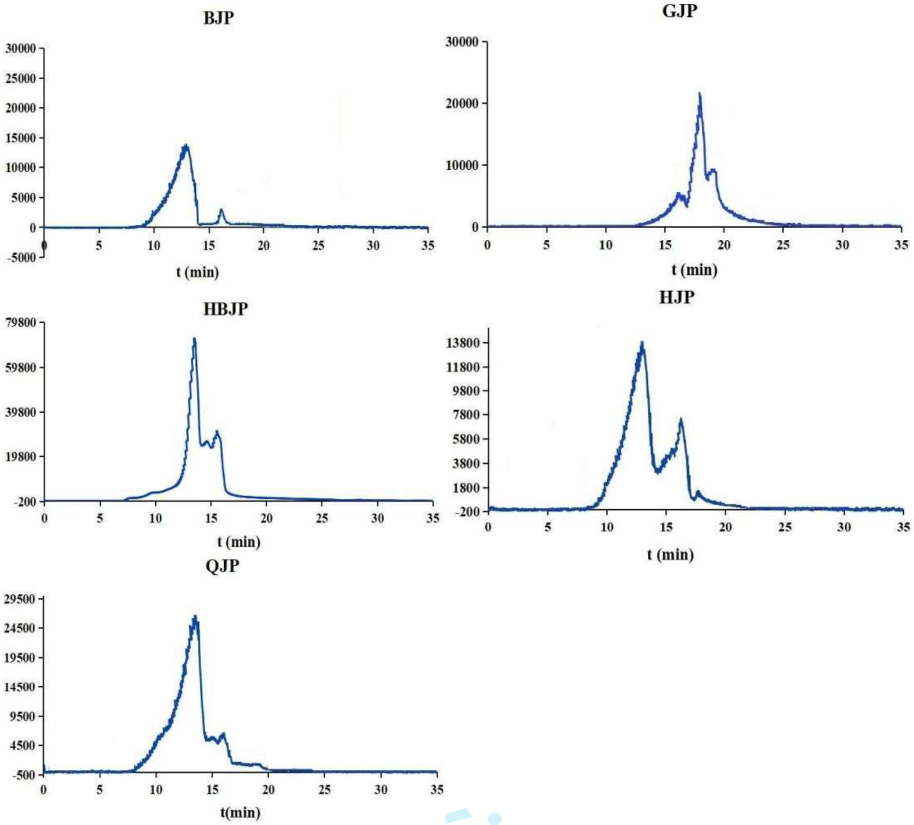

Fig. S3 High performance size exclusion chromatograms of BJP, GJP, HJP, QJP, and HBJP.

view Only

Table S1. Geographical and climatical information of the 5 populations of chrysanthemum.

Name	Province	Location	Latitude (N)	Longitude (E)	AMT (° C)	MA (m)	AMSH (h)	AMP (mm)
HBJ	Zhejiang	Jiaxing, Tongxiang	30°28′-30°27′	120°17′-120°39′	15.8	5.3	1983.4	1193.8
GJ	Anhui	Huangshan, Shexian	29°30′-30°7′	118°15′-118°53′	16.4	400	1928	1477
BJ	Anhui	Bozhou	32°51′-35°05′	115°53′-116°49′	14.9	30.5	2184	831
HJ	Henan	Jiaozuo, Wenxian	35°10′-35°21′	113°4′-113°26′	14.5	692.7	2484	625
QJ	Hebei	Anguo	38°42′48″	115°33′30″	12.4	30.6	2457.6	555.3

HBJ: Hangbaiju, GJ:Gongju, BJ: Boju, HJ: Huaiju, QJ: Qiju, AMT: Annual mean temperature, MA: Mean altitude, AMSH: Annual mean sunshine hours, AMP: Annual mean precipitation.
